# Complete Genome Sequence of a Canine-Origin H3N2 Feline Influenza Virus Isolated from Domestic Cats in South Korea

**DOI:** 10.1128/genomeA.00253-12

**Published:** 2013-03-07

**Authors:** Seong-Jun Park, Bo-Kyu Kang, Hye-Young Jeoung, Hyoung-Joon Moon, Minki Hong, Woonseong Na, Bong-Kyun Park, Haryoung Poo, Jeong-Ki Kim, Dong-Jun An, Dae-Sub Song

**Affiliations:** a Viral Infectious Disease Research Center, Korea Research Institute of Bioscience and Biotechnology, Daejeon, South Korea; b Department of Veterinary Medicine Virology Laboratory, College of Veterinary Medicine and Research Institute for Veterinary Science, Seoul National University, Seoul, South Korea; c Research Unit, Green Cross Veterinary Products, Yongin, South Korea; d Animal, Plant and Fisheries Quarantine and Inspection Agency, Anyang, South Korea; e University of Science and Technology, Daejeon, South Korea; f Department of Pharmacy, College of Pharmacy, Korea University, Sejong, South Korea

## Abstract

A canine-origin Korean H3N2 feline influenza virus (FIV), A/feline/Korea/01/2010 (H3N2), was isolated in 2010 from a dead cat with severe respiratory disease. Here, we report the first complete genome sequence of this virus, containing 3′ and 5′ noncoding regions, which will help elucidate the molecular basis of the pathogenesis, transmission, and evolution of FIV.

## GENOME ANNOUNCEMENT

Influenza A virus (IAV), a member of the family *Orthomyxoviridae*, is an enveloped virus with eight segmented, negative-sense, single-stranded RNA genes. IAV is a clinically and economically important pathogen which infects many animals, including humans, pigs, horses, marine mammals, and poultry (1). Influenza had long been absent from the list of infectious diseases considered as possibilities in dogs and cats (2). However, this has been recently disproved by the isolation of IAVs in those animals (3–7). A canine-origin Korean H3N2 feline influenza virus (FIV) strain, designated A/feline/Korea/01/2010 (H3N2), was isolated at the beginning of 2010 from a lung specimen of a dead cat, which had suffered from a severe respiratory disease (6). To date, a complete genome sequence containing 3′ and 5′ noncoding regions (NCRs) of H3N2 FIV has not been reported despite multiple functions of NCRs in the replication of IAVs (8–11). Therefore, it is necessary to analyze the complete genome sequence containing both 3′ and 5′ NCRs of A/feline/Korea/01/2010 (H3N2) and elucidate its molecular characteristics.

Viral RNA was extracted from allantoic fluids of embryonated eggs infected with A/feline/Korea/01/2010 (H3N2) and circularized with T4 RNA ligase as described previously (12–15). To determine the complete genome sequence containing 3′ and 5′ NCRs, the PCR products produced by RNA ligation-mediated reverse transcriptase PCR (RT-PCR) were purified, cloned (16), and sequenced on an automated DNA sequencer (ABI System 3700; Applied Biosystems, Inc.) by utilizing universal primer sets (17) with slight modifications and newly designed segment-specific primers.

The complete genome of A/feline/Korea/01/2010 (H3N2) is 13,629 nucleotides (nt) long, a length identical to that of the avian-origin Korean H3N2 canine influenza virus, A/canine/Korea/01/2007 (H3N2) (13); segments 1 (Seg-1) through 8 (Seg-8) are 2,341, 2,341, 2,233, 1,765, 1,565, 1,467, 1,027, and 890 nt, respectively. They encode 12 viral proteins with amino acid lengths as follows: PB2, 759; PB1, 757; N40 (18), 718; PB1-F2, 90; PA, 716; HA, 566; NP, 498; NA, 469; M1, 252; M2, 97; NS1, 230; and NS2 (nuclear export protein [NEP]), 121.

While the lengths of the 3′ and 5′ NCRs of the viral RNA of A/feline/Korea/01/2010 (H3N2) were variable (19 to 45 and 20 to 58 nt at the 3′ and 5′ NCRs, respectively) in the different genome segments, the terminal 12 and 13 nt of the 3′ and 5′ ends, respectively, were highly conserved (3′-UCGYUUUCGUCC- and -GGAACAAAGAUGA-5′) among all eight genome segments, which is consistent with previous studies (13, 14, 19, 20). Surprisingly, 1 nt was changed between the start codon (UAC) and the conserved region (UCGYUUUCGUCC) in the 3′ NCR of Seg-1 and also in Seg-6 of A/feline/Korea/01/2010 (H3N2) compared to A/canine/Korea/01/2007 (H3N2).

This is the first report of the complete genome sequence containing the 3′ and 5′ NCRs of H3N2 FIV. We hope that these data will provide important insights into the molecular basis of the pathogenesis, transmission, and evolution of FIV as well as other IAVs.

### Nucleotide sequence accession numbers.

The complete genome sequence of the A/feline/Korea/01/2010 (H3N2) strain described here has been deposited in GenBank under the accession numbers KC422453 to KC422460 for Seg-1 to Seg-8.
